# Comparing Brain Responses to Moral and Semantic Violations

**DOI:** 10.3390/brainsci16040375

**Published:** 2026-03-30

**Authors:** Jian Meng, Demi Zhang, Yuling Zhong, Xiaodong Xu, Edith Kaan

**Affiliations:** 1 Department of Linguistics, University of Florida, Gainesville, FL 32611, USA; mengjian@ufl.edu (J.M.); zhang.yidan@ufl.edu (D.Z.); 2 School of Foreign Languages and Cultures, Nanjing Normal University, Nanjing 210098, Chinaxiaodong_x@njnu.edu.cn (X.X.)

**Keywords:** moral judgment, LPC, ERP, semantic violation, language processing

## Abstract

**Highlights:**

**What is the main finding?**

- Encountering a moral violation while reading short texts elicits an earlier posterior positivity in the event-related brain potential response compared with semantic violations, which showed a descriptively more frontal positivity.

**What is the implication of the main finding?**

- The findings support the view that the processing of moral deviations involves mechanisms that are partially different in nature and timing from those recruited for the processing of semantic oddities.

**Abstract:**

Background/Objectives: The processing and evaluation of behavior, actions or events that go against social (moral) norms can be assumed to operate on mental representations of the world and of how people typically behave. These mechanisms and representations may therefore be shared by the processing of meaning in general. The current study investigated whether the processing of deviations of morality can be distinguished from processing of semantic inconsistencies. Methods: Event-related brain potentials (ERPs) were recorded from English speakers while they read short written texts in English for comprehension. Texts contained words that constituted moral violations, semantic violations and neutral controls depending on the context, allowing for a direct comparison. Results: Using trial-based analyses, we found different ERP responses to semantic and moral violations: the moral violation elicited a long-lasting, posterior Late Positive Component (LPC) starting at around 300 ms, whereas the semantic violation elicited a positivity that started later and was descriptively more frontally distributed. Furthermore, the LPC amplitudes could be explained by the moral acceptance scores over and above plausibility scores, but not vice versa. Conclusions: The outcomes are compatible with the view that the processing of moral deviations engages at least some mechanisms that are different from the processing of semantic deviations.

## 1. Introduction

Being able to tell right things from wrong is integral to human functioning. Moral knowledge and judgment are also intricately tied to language, given that most of human communication is done with language. Studies on the effect of using a non-native language on moral judgment and reasoning illustrate these connections [1,2]. In addition, the processing and evaluation of morality can be assumed to operate on mental representations of the world and of how people typically behave. These mechanisms and representations may therefore be shared by the processing of meaning in general in written and spoken discourse. In the current ERP study, we used short written texts and investigated whether the processing of deviations of morality can be distinguished from processing of deviations in semantic meaning, using direct comparisons, by-trial analyses, and without an explicit judgment task. To preview our findings, we found different ERP responses to semantic and moral violations, suggesting that the processing of moral deviations engages at least some mechanisms that are different from the processing of semantic deviations.

### 1.1. Moral Processing and ERP Components

In this paper, we define moral violations as behavior, actions or events that go against social norms, ranging from killing, stealing and incest to laughing at someone’s sorrow, or swearing in a church. Moral judgment has been proposed to involve several underlying mechanisms, including an affective and a normative component [3]. The affective process is typically associated with an initial emotional reaction to the moral stimuli, whereas the normative mechanism reflects a slower evaluative process. The two systems operate independently, but they can be engaged simultaneously [4] and even influence each other [5]. The affective process can be automatic or controlled [6]. That means while emotional reactions towards moral events can be naturally induced, higher-level cognitive processes, such as language, attention, and memory retrieval, can affect how the stimulus is perceived during one’s moral judgment process. These proposed mechanisms are supported by data from neuroimaging studies (e.g., [7]). In particular, moral judgment studies using event-related brain potentials (ERPs) have identified early ERP components that have been interpreted to reflect initial affective reactions and attention monitoring, such as the P200 [8,9]. In addition, later positive components have been observed that are taken to correspond to the later evaluative phase [8,9,10,11,12,13,14].

These later positive components are referred to as the Late Positive Potential (LPP), the Late Positive Component (LPC), or P300 [15]. It is still unclear to what extent these positivities are different components. Some studies report a frontal positivity starting around 300 ms and lasting until 500–600 ms [16,17]. An increase in amplitude of this positivity is interpreted as a reaction to morally inconsistent behavior, the evaluation of incongruent information related to the task, or reflecting emotional arousal [16]. A more consistent finding is a positivity starting at 200 ms to 500 ms, and lasting until 700 ms to 1000 ms, with a central or parietal scalp distribution [8,9,10,11,12,13,14]. We will refer to this posteriorly distributed positive component as the LPC in this paper. The LPC has been observed to be larger in response to immoral than moral behaviors and has been interpreted to reflect a more controlled evaluative activity [12], or a higher-order process in reaction to painful and harmful experiences [9,11].

Detecting and evaluating moral digressions of behavior can be assumed to operate on mental representations of the world and of how people typically behave. These mechanisms and representations may therefore be shared by the processing of meaning in general in written and spoken discourse. On the other hand, encountering moral digressions may evoke additional affective and emotional processes, or may engage unique processes altogether. Studying these similarities and differences may therefore provide further insight into the mental architecture involved in processing moral digressions.

Results from ERP studies that directly or indirectly compare the processing of moral digressions with that of semantic deviations are inconsistent, however. One study [8] compared texts ending in sentences that had words inducing moral inconsistencies, and words that induced a semantic inconsistency. A P200 effect was elicited for both types of inconsistency (vs. their consistent counterparts), suggesting that early attentional processes are shared between moral and semantic processing. A different study [18] also found a smaller P200 for words that expressed an emotion that was inconsistent versus consistent with the preceding scenario (e.g., somebody feeling flattered vs. desperate after hearing his family may have been in a flood) but did not report a difference in P200 for semantic inconsistencies versus controls. Other studies do not report P200 effects for moral inconsistencies vs. control conditions in written texts [1].

Explicit moral judgment studies using written text [1,19] found a larger N400 amplitude for words that induced a moral inconsistency with the participant’s or common values compared to words that did not (e.g., *The fact that African children die of starvation is catastrophic/beneficial*…, [1]). The N400 component (300–500 ms) is typically larger for violations of word knowledge and meaning versus expected words. This component has been proposed to reflect lexical activation and integration: a larger N400 amplitude reflects less pre-activation and harder integration into the context [20]. Although these moral judgment studies [1,19] did not include any semantic violation conditions in their study, the N400 effect suggests an overlap between the processing of moral and semantic inconsistencies in terms of lexical retrieval and integration. Studies that include violations of social normative behavior/emotions as well as semantic violations report an N400 effect for both violation types [18,21,22,23], with the N400 effect being smaller for moral than for semantic anomalies [18,23] or with a distribution being differentially affected by the participants’ cultural environment [22]. However, two studies [8,24] report an N400 effect only for words that constituted a semantic violation versus control words, but not for words that induced a violation of social norms.

Finally, findings concerning the LPC are inconsistent as well. One study [7] reports an LPC for moral violations but not for violations of world knowledge. This suggests that words that denote moral digressions induce late evaluative or affinitive processes that are different from processes dealing with semantic inconsistencies. However, others [18,24] do not report an LPC for either semantic or moral violations versus their controls or report a frontal-distributed positivity for moral violations [23]. Studies that do not directly compare moral and semantic violations show inconsistencies as well: some studies report an LPC for moral inconsistencies [1,19], some do not [11], and some find a frontally distributed positivity [21].

We should note that observing an N400 or LPC may be complicated by component overlap. The two components have opposite polarities and overlapping posterior scalp distributions. An LPC may therefore obscure an N400 effect [8,10], although some studies report both N400 and LPCs for moral inconsistencies [1,19].

### 1.2. The Current Study

It is therefore still rather unclear how the processing of words expressing morally objectional behavior differs from processing words that express situations that do not make sense given what we know about the world. To our knowledge, only few studies [8,18,22,24] have directly compared the ERP components in response to semantic and moral violations. In addition, a few studies in moral processing have used explicit judgments, which may lead participants to recruit processes and strategies not typically used [1,19,22,24]. The current study investigated English speakers’ processing of moral violations and how this differs from the processing of semantic violations. We presented readers with brief written texts consisting of a context sentence and a target sentence. The type of violation was manipulated by varying the context sentence. The target sentence, including the target word giving rise to the violation, was the same across conditions. This allows us to closely compare the processing of semantic and moral violations, and a neutral control. This is different from previous research [8,18,22,24] which used different target words for semantic and moral violations. Participants read the passages for comprehension, not for the purpose of judging acceptability, in contrast to some previous studies [1,11,19,22,24]. If detecting and processing moral violations is different from detecting and processing semantic violations, we expected to see differences in the ERP components elicited. In particular, if moral violations draw more attention and evoke evaluative processes, we expected a larger P200 and LPC for the moral versus semantic violation conditions. If words constituting semantic violations are harder to retrieve and integrate into the preceding context than words that signal moral digressions, the semantic violation conditions will give rise to a larger N400 than moral violations [18,21]. Another difference with prior studies is that we conducted by-trial analyses to assess the LPC’s sensitivity to moral acceptability. If the LPC is reflecting the response to moral inconsistencies rather than to semantic oddities in general, we expected this component to vary as a function of morality judgment of the item over and above its plausibility judgement, but not vice versa. Finally, we expected the LPC to vary with the individual participant’s moral acceptability of the item obtained after the EEG study.

## 2. Materials and Methods

### 2.1. Participants

We recruited a total of 38 monolingual English speakers at the University of Florida, USA. This number was based on prior studies [7,17]. Informed consent was obtained from all participants. Participants received either monetary compensation (US$20) or extra course credits. The inclusion criteria for participants were that English was their first language, and they did not speak another language in their household growing up; they were right-handed, had no reading or hearing difficulties, and were neurologically healthy as per self-report. One participant reported having experienced head injury and seizure in the past. However, removing their data did not show a significant difference in the results of these analyses (DFBETA absolute values smaller than the threshold value for both fixed-effect slopes), so we retained their data in the analyses reported below. Data of five participants were rejected from analysis for having fewer than 60 out of 90 artifact-free epochs (see Section 2.4), leaving a total of 33 datasets (23 women, 9 men, 1 non-binary; mean age 21.2 years (SD = 2.75)).

### 2.2. Materials

#### 2.2.1. Conditions

We initially created a set of 120 item sets. Each set had three versions corresponding to the three conditions in the study: moral violation, semantic violation, and normal condition. An example of the three conditions is shown in Table 1. An item consisted of a context sentence followed by a target sentence. The target sentence was always the same across the three conditions in the item set, but the context differed. The target sentence contained a critical word (e.g., “sped up”, bolded in Table 1) that gave rise to a moral violation (a), semantic violation (b), or was neutral (c) given the context. In the moral violation condition, the critical word introduced an action or situation that is not moral, for instance speeding up a car when children are crossing. In the semantic violation condition, speeding up the car is not semantically plausible since the car had no gas. In the neutral condition, the target was a plausible continuation of the context sentence.

#### 2.2.2. Norming

The 120-item triplets were pre-tested in a norming task. A total of 68 native English speakers were recruited through Qualtrics (https://www.qualtrics.com) to judge the plausibility (very implausible/unlikely 1 — very plausible/likely 5) of the items. Participants saw the context and the target sentences, but the target sentences were cut off just after the critical word (e.g., “so she sped up…”). Each item (version) was rated by at least 11 participants. In an additional study, 96 different participants were presented the items in the same format but were asked to rate on a 5-point Likert scale how morally acceptable (with 1 corresponding to “very immoral”, and 5 to “very moral”) and how emotionally moving (1 being “not emotional”; 5 “very emotional”) they considered the items, and were asked to judge the valence of the emotion (ranging from 1 “negative” to 5 “positive”).

On the basis of the norming tests, 90-item sets were selected such that the moral violation version was judged the least moral and the most emotionally moving, the semantic violation version had the lowest plausibility scores, and the neutral condition had the highest plausibility scores. One-way ANOVAs showed that the moral scores statistically differed across the conditions [moral scores: F(2, 267) = 427.1, *p* < 0.001]: the moral acceptance scores for the neutral condition were the highest among the three conditions, and the lowest for the moral condition (neutral: M = 3.72, SD = 0.51; semantic: M = 2.81, SD = 0.55; moral: M = 1.62, SD = 0.37). Post hoc Tukey tests showed a significant difference between all three conditions (ps < 0.0001; mean difference: moral vs. neutral −2.11 95% CI [−2.28, −1.95]; semantic vs. neutral −0.91, 95% CI [−1.09, −0.74]; semantic vs. moral 1.19, 95% CI [1.02, 1.36]). Plausibility scores also statistically differed across the conditions [one-way ANOVA: F(2, 267) = 618.4, *p* < 0.001], with the neutral condition being the most plausible among the three conditions, and the semantic condition rated the least plausible (neutral: M = 4.70, SD = 0.24; semantic: M = 1.64, SD = 0.49; moral: M = 2.73, SD = 0.87; ps < 0.0001 for all post hoc Tukey comparisons. Mean difference: moral vs. neutral: −1.98, 95% CI [−2.18, −1.77]; semantic vs. neutral: −3.06, 95% CI [−3.27, −2.85]; semantic vs. moral: −1.08, 95% CI [−1.29, −0.87]). Materials and norming data are available at https://osf.io/gquje/ (accessed on 15 March 2026).

#### 2.2.3. Surprisal Values

To assess potential differences among the conditions as to the predictability of the critical word, we obtained surprisal values as a proxy. We quantified lexical predictability using surprisal values derived from OpenAI’s GPT-3 model (davinci−002), with 175-billion parameter version [25] accessed through the OpenAI API (https://platform.openai.com/) (v1/completions; accessed on 15 April 2025). The surprisal for a target word w_t in context w_{ < t} was calculated as S (w_t) = −log_2_ P (w_t|w_{ < t}) [26]. While GPT-3 natively returns log probabilities using the natural logarithm, we converted these to base 2 for consistency with standard information-theoretic practice. The adapted scripts for surprisal value calculation is available online [27].

The 270 stimuli (90 per condition) were processed through the OpenAI API using the Completion endpoint with two critical parameters: logprobs =1 (to return token-level log probabilities) and echo = True (to return probabilities for the input text). To ensure accurate alignment between target words and GPT-3’s subword tokenization, we implemented a verification step using the GPT-2 tokenizer from HuggingFace’s transformer library. A one-way ANOVA revealed a significant main effect of condition on surprisal scores, F(2, 266) = 44.29, *p* < 0.001. Post hoc Tukey tests indicated that surprisal scores were significantly higher for semantic violations (M = 12.04, SD = 4.78) compared to both moral violations (M = 8.14, SD = 4.09; mean difference = 3.91, 95% CI [2.44, 5.37], *p* < 0.001) and neutral sentences (M = 6.32, SD = 3.52; mean difference = 5.73, 95% CI [4.26, 7.19], *p* < 0.001). Additionally, moral violations had significantly higher surprisal values than neutral sentences (mean difference = 1.82, 95% CI [0.35, 3.29], *p* = 0.010).

#### 2.2.4. List Creation

The 90 experimental items obtained as described in the above were distributed over 3 lists using a Latin Square design. We created 30 filler items, which were the same for each list. These filler items were similar to the neutral condition in that they contained a context sentence followed by a target sentence that did not contain any violations. Each list had 30 items for each of the three experimental conditions, and 30 fillers. Each list was divided into 5 blocks, each containing 24 items. The blocks included an equal number of experimental items and filler items from all three conditions. Within each list, 48 experimental items and 16 fillers (53% of the sentences) were followed by comprehension questions. The comprehension questions were simple and fact-based. For example, for the item “Mary found out her boss Todd had an affair. Today she was fired from her job.”, a comprehension question would be “Did Mary keep her job?”. Questions only served the purpose of attention checks and did not systematically probe the violations contained in the items. During the experiment, participants were assigned to one of the lists.

### 2.3. Procedure

All participants gave informed consent. Participants then completed a health questionnaire, a handedness questionnaire [28], and a language background questionnaire to report the languages the participant spoke and the proficiency level.

For the EEG experiment, the participants were fitted with an elastic electrode cap (see Section 2.4), and were seated in an electrically shielded, sound-attenuating booth. They were positioned about 1 m from a computer monitor. Participants used a game console to press for the next sentence or answer reading questions, using the left trigger for “No” and the right trigger for “Yes”. Stimulus display and behavioral data collection were conducted through Eprime 2.0 pro and 3.0 (Psychological Software Tools, Pittsburgh, PA, USA). The stimuli were presented in silver Courier New 24 pt on a black background. Before each item, a fixation cross appeared on the screen for 500 ms. The participants would then see the entire context sentence, which remained on the screen until the participant pressed the button indicating they had finished reading. After an 800 ms blank screen, the critical sentence appeared on the screen segment by segment each for 400 ms, separated by a 200 ms blank screen. An 800 ms blank screen appeared after each sentence, which was replaced by either a “press for next” message or a comprehension question. The comprehension questions appeared randomly in 13 of the trials in each block, with one block only having 12 questions. Each block lasted about 7 min.

After the sentence processing task, subjects were assigned a Qualtrics questionnaire to report their judgment on the 30 moral violation items they had seen in the study, among 30 neutral sentences. Items were cut off after the critical word. Participants indicated on a 7-point Likert scale how morally accepting (1 being very immoral and 7 being very moral) and how emotionally moving the items were (1 being not emotional and 7 being very emotional). Participants also completed the 32-item moral disengagement questionnaire [29]. We did not observe any effects of this latter measure; therefore, we do not report these data.

### 2.4. EEG Acquisition and Analysis

EEG data were collected using ANT-Neuro Waveguard^TM^ elastic caps with 32 scalp electrodes positioned according to the 10–20 system (to see detailed locations of the electrodes, refer to [30]). Additionally, eye movements and blinks were tracked through bipolar electrodes placed beside both eyes and above and below the right eye. Additional electrodes were placed on the left and right mastoids. The impedance levels of the electrodes were maintained below 5 kOhm. EEG data were collected using an ANT Refa 78 amplifier (ANT-Neuro, Hengelo, The Netherlands) with a rate of 512 Hz and referenced to AFz.

The analysis of the EEG data was conducted in EEGLAB [31] with the ERP lab plug-in [32]. EEG signals were filtered with a high-pass filter set to 0.1 Hz and a low-pass filter at 30 Hz. Then, the data was re-referenced to the mean amplitudes of two mastoids. Noisy data typically caused by muscle movements at the beginning and the end of the blocks were removed manually from the raw data. After this, ICA were performed using runica in EEGLab. We then removed 2 to 3 components on average, reflecting horizontal and vertical eye movements from each participant’s data, which were identified through IClabel [33]. Noisy channels were interpolated. The number of channels interpolated for each participant ranged from 0 to 4 (typically T7 and T8 were interpolated).

We then extracted epochs from −200 to 1200 ms related to the onset of the critical words. Baseline correction was applied by subtracting the mean voltage of the pre-stimulus interval (−200 ms to 0 ms) from all time points within each epoch. Simple voltage threshold and moving window peak-to-peak were performed for artifact rejection on the epochs. For simple voltage threshold, trials that exceeded ±75 μV were rejected. For certain participants, this criterion was adjusted to ±100 μV. The length of the moving window was 200 ms and the window step was 100 ms. The voltage threshold was 75 and was adjusted to 100 μV for certain participants. All rejections were manually checked. Fp1, Fpz, Fp2, T7, T8, O1, Oz, and O2 were left out for this analysis. Participants with more than 2/3 of trials identified as artifacts were excluded from further analysis (*n* = 5). The rest of the subjects (*n* = 33) all had at least 60% usable trials per condition, which was 18 out of 30 trials.

ERP amplitudes for three critical time windows were extracted for each participant, item, electrode and condition. The P200 was defined as the mean between 150 and 250 msc [12] after target word onset across the frontocentral electrodes: FCz, FC1, FC2, FC3, FC4, Cz, C3, and C4. The N400 was quantified as the mean amplitude between 300 and 500 ms after target word onset across the centroparietal electrodes: Cz, C3, C4, CP1, CP2, CP5, and CP6; the late potentials as the mean amplitude between 500 and 900 ms after onset averaged across the parietal electrodes CP1, CP2, CP5, CP6, Pz, P3, P7, P4, P8, and POz [8].

A linear mixed-effects model using the lme4 package version 1.1–21 [34] in R version 4.4.2 [35] was estimated for each of the three trial-based ERP amplitudes as defined above. Condition was the fixed effect. This was Helmert coded to test two contrasts. The first (violation contrast) compared violation (moral and semantic violation conditions) versus the neutral condition. The neutral condition was coded as negative (−2/3) while the violation condition was coded as positive (1/3 for moral and 1/3 for semantic condition); the second contrast compared the two violation types. The moral condition was coded as positive (1/2) while the semantic condition as negative (−1/2). Our initial model included by-subject and by-item intercepts and condition as by-subject and by-item slopes. This model yielded singularity issues. We then reduced the random-effect structure by dropping the random-effect with the smallest variance. The final models included only by-subject and by-item random intercepts. These final models did not differ from the models with a maximum random-effects structure in terms of which effects were significant and which were not. Data, scripts and complete model outcomes are available at https://osf.io/gquje (accessed on 15 March 2026).

## 3. Results

### 3.1. By-Window ERP Analysis

**Figure 1:**
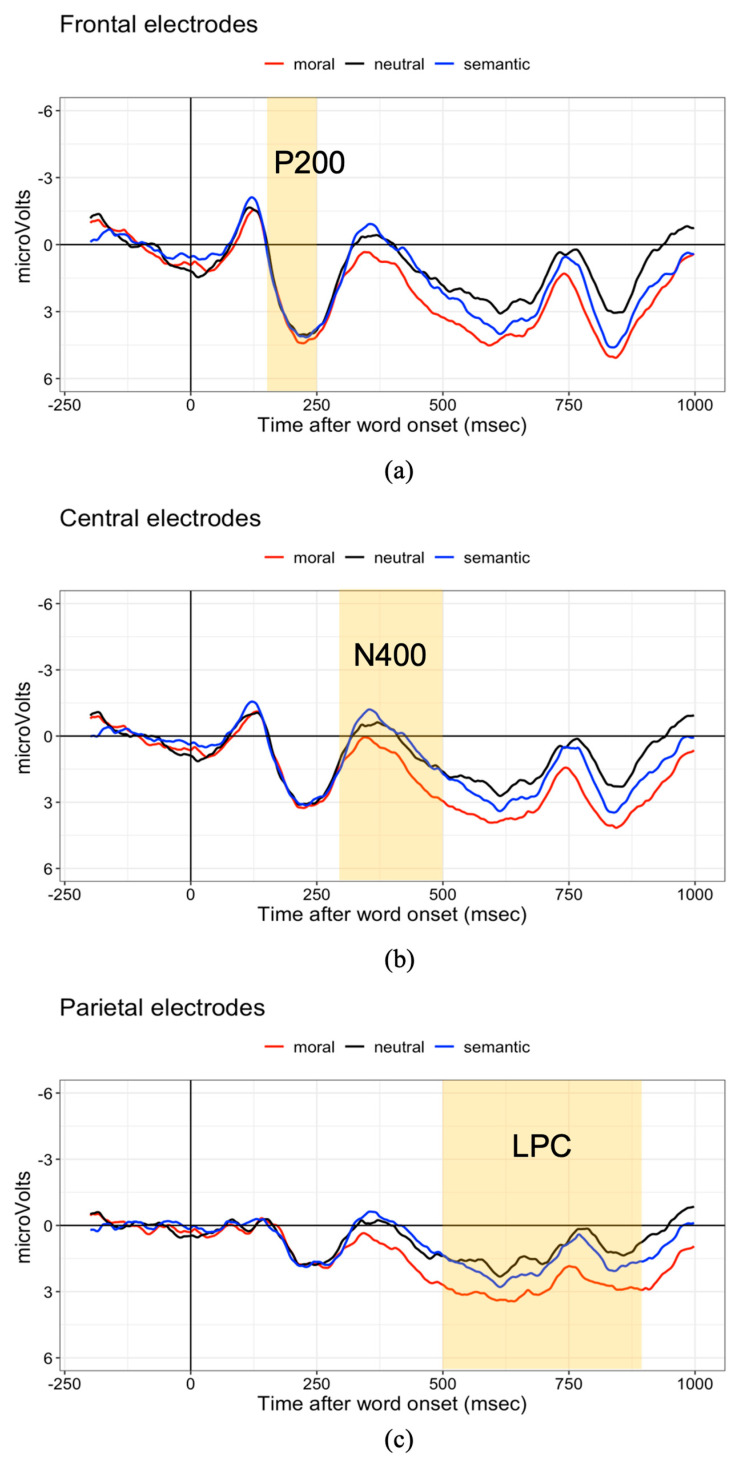
Mean ERPs at the critical word. The red line refers to the moral violation condition; the black line refers to the neutral (no-violation) condition; the blue line refers to the semantic violation condition. The highlighted yellow areas are the time windows analyzed. (**a**): ERP amplitudes averaged over frontal sites (FCz, FC1, FC2, FC3, FC4, Cz, C3, C4) to assess the P200. (**b**): ERP amplitudes averaged over the central sites (Cz, C3, C4, CP1, CP2, CP5, CP6) to assess the N400. (**c**): ERP amplitudes averaged over the parietal sites (CP1, CP2, CP5, CP6, Pz, P3, P7, P4, P8, POz) to assess the LPC.

The average percentage of correct responses to the comprehension questions was 97% (range 86–100%), suggesting that our participants were attentive. No participant was excluded for low comprehension scores. Mean ERPs time-locked to the critical word for the frontocentral, central and centroparietal electrodes are shown in Figure 1.

#### 3.1.1. P200

No significant results were found for the 150 ms to 250 ms (frontocentral electrodes) window. The ERP amplitudes showed no statistical differences between the neutral condition and the two violation conditions (b = 0.14, 95% CI [−0.44, 0.72], SE = 0.30; T = 0.47, *p* = 0.64), or between the two violation conditions (b = 0.28, SE = 0.34, 95% CI [−0.39, 0.95]; T = 0.81, *p* = 0.42).

#### 3.1.2. N400

When examining the N400 window (300 ms to 500 ms) at the central electrodes, we found a difference between the semantic and moral violation condition in that the ERPs for the moral violation condition were more positive than for the semantic condition [b = 1.16, 95% CI [0.48, 1.85], SE = 0.35; T = 3.35, *p* < 0.001]. No difference was found between the two violation conditions taken together and the neutral condition (the violation contrast) [b = 0.20, 95% CI [−0.40, 0.79], SE = 0.30; T = 0.65, *p* = 0.52]. We had expected the N400 to be larger for the semantic violation than for the neutral condition, but the ERP traces did not visually differ for these conditions in the N400 time window. To assess this, we constructed a linear mixed-effects model that was the same as the main model, but with contrasts comparing the semantic and moral condition each versus the neutral condition; this yielded no significant difference between the amplitudes of neutral and semantic violation conditions [b = −0.38, 95% CI [−1.07, 0.30], SE = 0.35; T = −1.10, *p* = 0.27].

As a sanity check, we tested whether the N400 in our materials was sensitive to surprisal (predictability). To this aim, we conducted an analysis in which LLM surprisal was included as a continuous (centered) factor without the factor condition, but including by-subject and by-item random intercepts. The results showed that the N400 amplitude was more negative with larger surprisal (b = −0.12, SE = 0.03, T = −3.39, *p* < 0.001), suggesting that the N400 is indeed sensitive to predictability in our study (see Supplementary Material: Statistical Model Outputs).

#### 3.1.3. LPC

**Figure 2:**
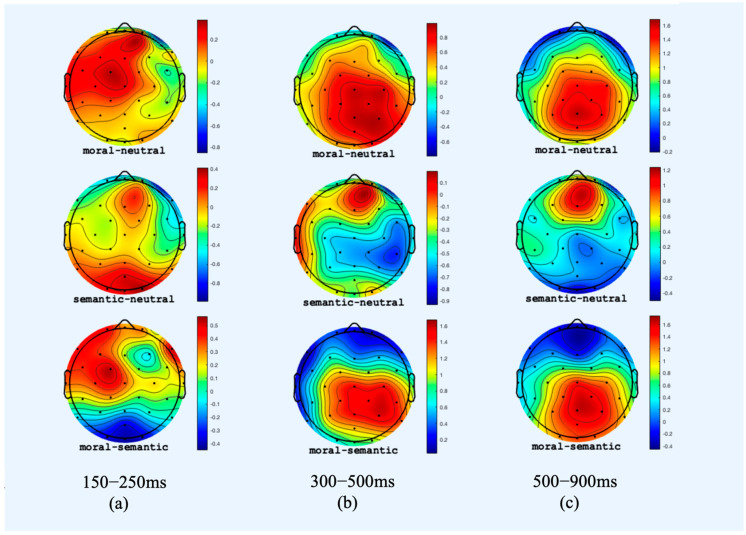
Topomaps of three time windows. The red indicates a more positive difference: isovoltage maps for the average amplitude for each of the three highlighted time windows for the moral minus neutral condition (**top row**), semantic minus neutral condition (**central row**), and moral minus semantic condition (**bottom row**). (**a**) Topograph for the 150–250 ms window. (**b**) Topograph for the 300–500 ms window. (**c**) Topograph for the 500–900 ms window.

In the 500–900 ms window (central–parietal electrodes), the two violation conditions showed a significantly larger LPC amplitude than the neutral condition [b = 0.74, 95% CI [0.18, 1.30], SE = 0.29; T = 2.59, *p* < 0.01]. The ERPs for the moral condition had a more positive amplitude than for the semantic condition [b = 1.10, 95% CI [0.46, 1.75], SE = 0.33; T = 3.35, *p* < 0.001], which is likely a continuation of the effect seen in the N400 window. Note that visually, the LPC for the semantic condition had a more frontal distribution, whereas the LPC for the moral violation was more posterior; see Figure 2c. To explore differences in scalp distribution, we built an lmer adding location as a fixed effect to the main model (frontal–central sites: FCz, FC1, FC2, FC3, FC4, Cz, C3, and C4 versus central–parietal sites: CP1, CP2, CP5, CP6, Pz, P3, P7, P4, P8, POz). Condition was coded such that the semantic and moral violation conditions were each compared to the neutral conditions. No interaction of location by condition was observed; however, the following results were observed: neutral vs. semantic by location: b = −0.44, SE = 0.50; T = −0.88; *p* = 0.38; neutral vs. moral by location: b = −0.05; SE = 0.49; T = −0.10; *p* = 0.92.

### 3.2. LPC and Moral Violations

The goal of our study was to directly compare the effects of moral and semantic violations on the ERPs. To assess to what extent the posterior LPC effect reflects the processing of moral violations rather than violations of semantic knowledge, we conducted two additional analyses on the ERP data. First, we investigated to what extent the LPC effect could be explained by the moral acceptance score beyond plausibility. Second, we investigated to what extent the LPC would be sensitive to the individual’s moral acceptance of the sentence.

For the first analysis, we used the moral acceptability scores and plausibility scores collected in the norming studies. A Pearson’s correlation test showed that the moral acceptance scores and the plausibility scores were correlated [r = 0.49, *p* < 0.0001]. We constructed three linear mixed-effects models using the mean LPC amplitudes (by-trial amplitude between 500 and 900 ms collapsed over parietal electrodes) as the dependent variable, and by-subject and by-item random intercepts. Instead of the condition factor, we entered moral acceptance scores (centered) as the fixed effect in the first model (moral score model), plausibility scores (centered) in the second model (plausibility model), and both in the third (full) model. We then compared the moral score and plausibility model against the full model using the anova function. The results showed that while the absence of the moral acceptance scores leads to a significant difference with the full model [χ^2^ = 18.84, *p* < 0.0001], the absence of the plausibility scores did not [χ^2^ = 2.10, *p* = 0.15]. This suggests that the LPC at the posterior electrodes could be explained by the moral acceptability scores over and above plausibility ratings, but not vice versa, in spite of the correlation between plausibility and acceptability scores.

In the second analysis, we used the participants’ own judgments of the moral items they read in the study. We collected these scores right after the ERP study. Participants were asked to judge how morally acceptable and how emotionally moving the moral condition stimuli were. These two scores were highly correlated (Pearson’s r = 0.49, T = −19.19, *p* < 0.001). Therefore, we only selected the moral acceptability score as the fixed effect. A linear mixed-effects model was constructed on the mean LPC amplitudes for the moral violation items only. This analysis was done on 32 datasets, since the post-EEG moral acceptability scores were missing for one participant. The model included by-participant and by-items random intercepts. Results showed that with lower moral acceptability scores, the LPC amplitude was larger, although this effect did not reach significance [b = −0.38, 95% CI [−0.87, 0.12], SE = 0.25; T = −1.50, *p* = 0.14] (see Supplementary Material: Statistical Model Outputs). We also tested the positivity for the moral condition in the 300–500 ms window. If this is an early LPC, as we hypothesize, we would expect this earlier positivity to vary with individual moral acceptability scores as well. Numerically, this was the case: with lower moral acceptability scores, the amplitude at central electrodes became more positive in the 300–500 ms window, but this was not statistically significant [b = −0.39, 95% CI [−0.92, 0.13], SE = 0.27; T = −1.47, *p* = 0.14].

## 4. Discussion

The current ERP study investigated the differential processing of moral versus semantic violation in language contexts. In contrast to prior studies, we kept the target words and sentences the same across the three conditions and varied the preceding context sentence. We did not find any effect in the P200 window but did find significant differences in ERP amplitudes between the semantic violation and the moral violation condition in both 300–500 ms and 500–900 ms windows. The moral violation elicited a larger positivity than the semantic condition in both windows. The 500–900 ms positivity was posteriorly distributed for the moral violation condition (LPC), while the positivity for the semantic violation was descriptively more frontal. The LPC amplitudes could be explained by the moral acceptance scores over and above plausibility scores, but not vice versa, suggesting that the LPC reflects mechanisms related to moral processing that are not shared by the processing of semantic oddities. In the below, we will briefly discuss why we did not observe P200 effects, why we did not find an N400 effect, and discuss the LPC.

### 4.1. P200

We failed to find an effect of violation or violation type on the P200 component. The P200 has been associated with affective processing and attentional shift [9,12]. One explanation for the lack of P200 effects is the nature of our stimuli and our task. Note that in our materials, the critical word only becomes a moral or semantic violation when integrated into the preceding context. This is in contrast with the study [19] in which the researchers report a P200, but used critical words that had strong emotional connotations even in isolation (such as “wrong/fine”, “bad/good”, and “forbidden/allowed”). Furthermore, in our study, participants were reading for comprehension rather than explicitly evaluating the stimuli. In order to tap into early affective processing and observe the P200 effect, more emotionally explicit materials or tasks might be needed (but see [8] for finding P200 in a paradigm similar to ours).

### 4.2. N400

We did not find an N400 for the semantic versus neutral condition, even though the latter was rated as more plausible and had lower surprisal values. We should note that surprisal did have an effect when treated as a continuous factor, with larger N400 amplitudes for the items that had a higher surprisal value. Given that the surprisal scores overlapped between the neutral and semantic violation conditions, a possible explanation is that N400 differences in the main analysis may have been obscured by the condition grouping (see the model output file on osf for a figure). In addition, as mentioned above, the target word gave rise to an anomaly only through the integration of the target sentence with the preceding context. This may have made the N400 more variable and less visible than in typical N400 studies (but see, e.g., [36]). Another reason why we did not find an N400 difference between the neutral and semantic violation conditions may be component overlap: the late positivity for the semantic violation condition may have overlapped with a potential in the N400 effect. To test this, we subtracted the averaged amplitudes of the neutral condition from the averaged semantic violation condition at the 300–500 ms window (central electrodes) and made the same subtraction at the 500–900 ms window (central–posterior electrodes). The size of the difference between the semantic violation vs. neutral condition was positively correlated between these two time windows (Pearson’s r = 0.66, T = 4.92, *p* < 0.001); that is, a smaller N400 effect (less negative amplitudes for semantic violations vs. neutral) was associated with a later late potential effect (more positive amplitudes). This is supportive of component overlap.

We also did not observe an N400 effect for the moral violation condition. Instead, we found an early onset of the posterior positivity for the moral condition rather than an N400. This contrasts with studies [18,19,21,22,23] that found larger N400 amplitudes for morally inconsistent versus consistent conditions. Quite a few previous studies report a positivity instead of a negativity in the N400 time window, however [8,10,11]. The absence of an N400 effect in the moral conditions in our study could have been a result of overlap with the late positivity. Note that the scalp distribution of the ERPs for the moral vs. neutral condition in the 300–500 ms and the 500–900 ms windows were almost identical in our study (see Figure 2). In addition, the difference in amplitudes between the moral violation and the neutral conditions were strongly positively correlated between the 300–500 ms window (central electrodes) and the 500–900 ms time windows (central–parietal sites) (Pearson’s r = 0.65, T = 4.71, *p* < 0.001). This suggests that the positive component for the moral conditions started at around 300 ms (see [11,19]), completely obliterating a potential N400 (negative-going) effect.

### 4.3. Late Positivities

The most important finding of our study is that we find a long-lasting posterior positivity (LPC) for the moral violation condition which started at around 300 ms, and a later, numerically more frontal, positivity for the semantic violation condition (although this location difference was not statistically supported). In the sentence processing literature, the late frontal positivity has been associated with updating processes after a strong prediction violation [37,38]. This frontal component is seen when expectation violations are embedded in rich, multi-sentence contexts compared to isolated sentences [39,40]. Words that induce a semantic anomaly (as in, e.g., the lifeguards […] cautioned the drawer, [37]) tend to elicit a late posterior positivity, which has been interpreted to reflect repair processes [37]. Most of the semantic inconsistencies in our study were not outright semantically anomalous but were implausible given the context (e.g., driving a car without gas). The frontal positivity we observe for our semantic violation conditions can therefore be interpreted as dealing with a violation of expectation and updating of the mental representations of the discourse (situation model). Note that the scenarios expressed in the moral violation conditions were not outright impossible, either, and were rated as significantly more plausible than the semantic violation conditions. The fact that the moral violation conditions elicited a posterior component suggests that these types of violations trigger processes and/or representations that are different from simply updating the mental discourse representation. The posterior LPC found for immoral or emotional stimuli has been interpreted as reflecting an evaluative process [8] or a sign of affective processing [11]. Supporting the latter interpretation is the finding that the LPC is larger for negative and highly arousing words [41,42]. We should note that our moral ratings were highly correlated with ratings as to the strength of emotions, so our findings are compatible with either the evaluative or affective interpretation of the LPC. The positivity started at around 300 ms, suggesting that these evaluative/affective processes started rather quickly after word recognition, whereas the process of updating in the semantic violation condition occurred later. Processing moral violations in written text therefore engages mechanisms quite different in nature and timing from those recruited for the processing of semantic oddities.

We are however cautious in attributing the LPC to processes unique to moral violations or emotional stimuli. The LPC has been commonly observed in various kinds of paradigms. As we mentioned in the above, late posterior positivities have been found for semantic violations that are outright anomalous, without involving moral or emotional stimuli [37]. In these cases, the positivity has been interpreted as reflecting repair processes. Late positivities have also been observed for phenomena as diverse as syntactic anomalies or difficulties [43,44,45], irony [46], and code-switching [45,47]. The LPC therefore seems to be a very general response. To what extent this component can be teased apart into separate positivities with each being a reflection of a specific operation or representational domain is a matter of future research.

## 5. Conclusions and Further Research

Our current study investigated the processing difference between moral violation and semantic violation. We compared ERPs to physically the same words while varying the preceding context sentence. When words gave rise to a moral violation given the context, a posterior ERP effect (LPC) was seen that started already at 300 ms, whereas words that gave rise to semantic violation were descriptively characterized by a late frontal positivity. The primary implication of our findings is that the human brain processes moral violations distinctively from semantic violations, especially at later stages of processing. We do need to point out that the topographic differences between the semantic and the moral violation conditions was not statistically reliable and hence should be interpreted with caution.

The strength of our study (comparing physically the same words across the conditions) is also its limitation, however. The context manipulation may have introduced factors not under our control. Confounds such as differences in priming, predictability, or emotional salience could have contributed to the observed effects. The results from our current study are therefore tentative. Future research should investigate the effects of moral violations while tightly controlling both the context and the target sentence.

Another limitation is the sample size. Although our sample size (*n* = 33) is close to that of previous studies [7,17], our study may have been underpowered. The effects observed, or the failure to observe effects, should therefore be interpreted with caution.

We are also cautious in attributing the LPC to processes unique to moral violations. The LPC has been commonly observed in various kinds of linguistic and non-linguistic paradigms that do not have much in common with moral or affective processing. Future research using machine learning would be one way to test whether the LPC can be divided into subcomponents that each are differently sensitive to particular features of the input.

Finally, we acknowledge that responses to moral violations may differ between individuals and groups [9]. Given the effect of language and culture on the evaluation of morality, an interesting further exploration would be to compare first- and second-language speakers of varying proficiency and length of immersion in a second-language environment [1]. In addition, the current study measured the moral and semantic processing of neuro-typical adult groups. It might also be interesting to extend the population to neurodivergent and/or non-adult groups, who may differ in their language processing [48] and have been shown to differ in their moral violation processing [49].

## Figures and Tables

**Table 1:** Examples of the three conditions.

Conditions	Context	Target Sentence
Moral	As Rose was driving to work, some school children started crossing the road in front of her.	So| she| **sped up|** the car| immediately ^1^.
Semantic	Rose was preparing to go to work today but discovered that her car had no gas.	So| she| **sped up|** the car| immediately.
Normal	As Rose was driving to work, she realized she was going to be late.	So| she| **sped up|** the car| immediately.

^1^ The vertical bar represents the segmentation of the words in the EEG study. The critical word region is in bold for illustration purposes only.

## Data Availability

The original data presented in the study are openly available on the Open Science Framework at https://osf.io/gquje/ (accessed on 15 March 2026).

## Supplementary Materials

The following supporting information can be downloaded at: https://www.mdpi.com/article/10.3390/brainsci16040375/s1. https://osf.io/gquje/overview (accessed on 15 March 2026), Supplementary Material: Statistical Model Outputs.

## Abbreviations

The following abbreviations are used in this manuscript:

LPC	Late Positive Component
LPP	Late Positive Potential
ERP	Event-related Potential
P200	Positivity peaks around 200 ms post onset
N400	Negativity peaks around 400 ms post onset
