# Supplementary material for: Comparing Brain Responses to Moral and Semantic Violations
Source: Brain Sci. 2026 Mar 30;16(4):375. doi: 10.3390/brainsci16040375 (PMC13115281; doi:10.3390/brainsci16040375)
Supplement: Supplementary file 1 [file brainsci-16-00375-s001.zip › brainsci-4155897-supplementary.pdf]

Supplemental Material: Statistical Model Outputs

Date: March 15, 2026

Author: Jian Meng

Overview

This document contains the statistical outputs from the main analyses, plots for non-major analyses, and full models of the main analyses (maximal random effects structures) at the end of the file requested by the reviewers.

P200 analysis, frontal site

Parameters	*Estimate*	*95% CI*	*SE*	*t*	*p*
Intercept	3.08 ***	2.35,3.80	0.37	8.32	<0.001
Neutral.v. Violation	0.14	-0.44,0.72	0.30	0.47	0.639
Moral.v.Semantic	0.28	-0.39,0.95	0.34	0.81	0.417
**Random Effects**					
σ^2	51.94				
$\tau_{00\ Item\_number}$	0.97				
$\tau_{00\ Subject}$	3.39				
$N_{Subject}$	32				
N_{Item_number}	90				
Observations	2654				
Marginal R^2 / Conditional R^2	0.000 / 0.078				

* *p<0.05* ** *p<0.01* *** *p<0.001*

N400 analysis, central sites

Parameters	*Estimate*	*95% CI*	*SE*	*t*	*p*
Intercept	0.60	-0.18,1.37	0.40	1.50	0.133
Neutral.v.Violation	0.20	-0.40,0.79	0.30	0.65	0.518
Moral.v.Semantic	1.16 ***	0.48,1.85	0.35	3.35	**0.001**
**Random Effects**					
σ^2	54.66				
τ_{00} Item_number	2.43				
τ_{00} Subject	3.60				
N Subject	33				
N Item_number	90				
Observations	2715				
Marginal R^2 / Conditional R^2	0.004 / 0.103				

* *p<0.05* ** *p<0.01* *** *p<0.001*

N400 analysis, central sites, comparison to neutral

Parameters	*Estimate*	*95% CI*	*SE*	*t*	*p*
Intercept	0.60	-0.18,1.37	0.40	1.50	0.133
Semantic.v.Neutral	-0.39	-1.07,0.30	0.35	-1.11	0.268
Moral.v.Neutral	0.78 *	0.10,1.46	0.35	2.24	**0.025**
**Random Effects**					
σ^2	54.66				
τ_{00} Item_number	2.43				
τ_{00} Subject	3.60				
N Subject	33				
N Item_number	90				
Observations	2715				
Marginal R^2 / Conditional R^2	0.004 / 0.103				

* *p<0.05* ** *p<0.01* *** *p<0.001*

LPC analysis, posterior sites

Parameters	*Estimate*	*95% CI*	*SE*	*t*	*p*
(Intercept)	2.01 ***	1.35,2.67	0.34	6.00	**<0.001**
Neutral.v.Violation	0.77 *	0.05,1.48	0.37	2.10	**0.036**
Moral.v.Semantic	1.06 **	0.31,1.81	0.38	2.78	**0.005**
**Random Effects**					
σ^2	48.33				
τ_{00} Item_number	1.22				
τ_{00} Subject	2.57				
τ_{11} Item_number.ConditionLabelViol	0.94				
τ_{11} Item_number.ConditionLabelMorSem	2.64				
τ_{11} Subject.ConditionLabelViol	1.28				
τ_{11} Subject.ConditionLabelMorSem	0.16				
ρ_{01}	-0.18				
	-0.31				
	0.79				
	0.00				
N Subject	32				
N Item_number	90				
Observations	2632				
Marginal R^2 / Conditional R^2	0.007 / NA				

* *p<0.05* ** *p<0.01* *** *p<0.001*

LPC location analysis

Parameters	*Estimate*	*95% CI*	*SE*	*t*	*p*
Intercept	2.45 ***	1.80,3.10	0.33	7.39	**<0.001**
Neutral.v.Sem	0.40	-0.09,0.88	0.25	1.61	0.108
Neutral.v.Moral	1.30 ***	0.82,1.78	0.25	5.27	**<0.001**
location	-0.82 ***	-1.22,-0.43	0.20	-4.08	**<0.001**
Neutral.v.Sem x location	-0.44	-1.41,0.53	0.50	-0.88	0.378
Neutral.v.Moralx location	-0.05	-1.02,0.92	0.49	-0.10	0.920
**Random Effects**					
σ^2	55.15				
τ_{00} Item_number	2.05				
τ_{00} Subject	2.52				
N Subject	33				
N Item_number	90				
Observations	5430				
Marginal R^2 / Conditional R^2	0.008 / 0.084				

* *p<0.05* ** *p<0.01* *** *p<0.001*

The N400 amplitude has a negative correlation with the surprisal scores.

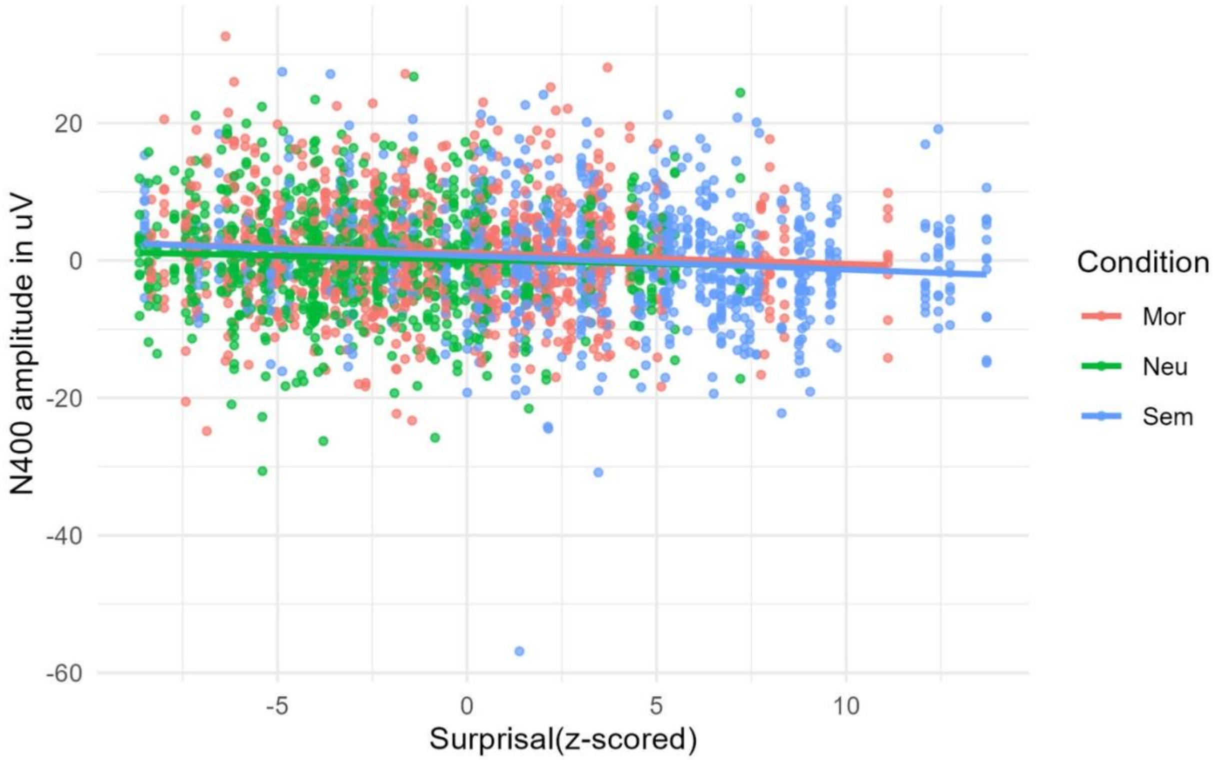

The surprisal scores for the neutral condition and the semantic violation condition shows overlaps.

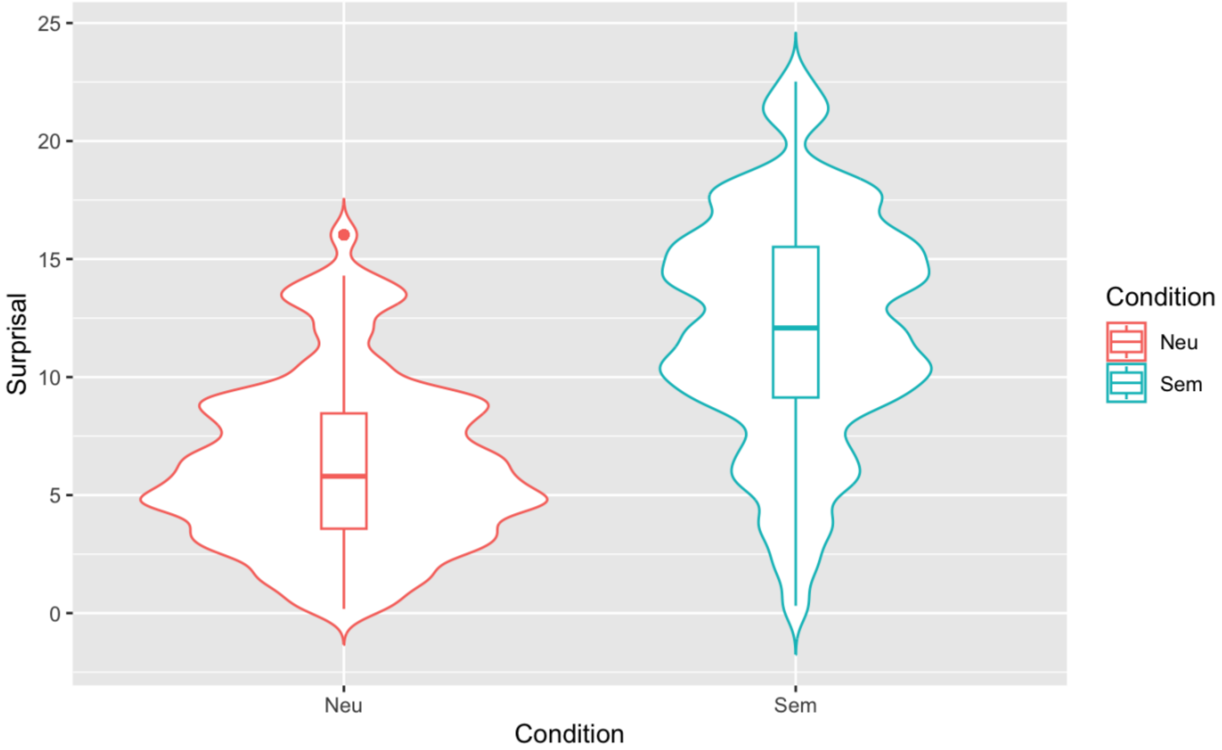

P200 analysis, frontal site (full model)

Parameters	*Estimate*	*95% CI*	*SE*	*t*	*p*
(Intercept)	3.08 ***	2.35,3.80	0.37	8.35	**<0.001**
Neutral.v. Violation	0.15	-0.52,0.82	0.34	0.45	0.656
Moral.v.Semantic	0.26	-0.53,1.06	0.41	0.65	0.517
**Random Effects**					
σ^2	51.08				
τ_{00} Item_number	1.02				
τ_{00} Subject	3.37				
τ_{11} Item_number.ConditionLabelViol	2.48				
τ_{11} Item_number.ConditionLabelMorSem	0.09				
τ_{11} Subject.ConditionLabelViol	0.08				
τ_{11} Subject.ConditionLabelMorSem	1.55				
ρ_{01}	-0.32				
	-0.92				
	1.00				
	-0.32				
N Subject	32				
N Item_number	90				
Observations	2654				
Marginal R^2 / Conditional R^2	0.000 / NA				

* *p*<0.05 ** *p*<0.01 *** *p*<0.001

N400 analysis, central sites (full model)

Parameters	*Estimate*	*95% CI*	*SE*	*t*	*p*
(Intercept)	0.59	-0.18,1.37	0.40	1.50	0.133
ConditionLabelViol	0.19	-0.42,0.81	0.31	0.62	0.536
ConditionLabelMorSem	1.16 **	0.41,1.92	0.39	3.02	**0.003**
**Random Effects**					
σ^2	54.32				
τ_{00} Item_number	2.46				
τ_{00} Subject	3.59				
τ_{11} Item_number.ConditionLabelViol	0.09				
τ_{11} Item_number.ConditionLabelMorSem	1.01				
τ_{11} Subject.ConditionLabelViol	0.20				
τ_{11} Subject.ConditionLabelMorSem	0.58				
ρ_{01}	-0.64				
	-0.97				
	0.43				
	0.64				
N Subject	33				
N Item_number	90				
Observations	2715				
Marginal R^2 / Conditional R^2	0.004 / 0.109				

* *p<0.05* ** *p<0.01* *** *p<0.001*

LPC analysis, posterior sites (full model)

Parameters	*Estimate*	*95% CI*	*SE*	*t*	*p*
(Intercept)	2.01 ***	1.35,2.67	0.34	6.00	**<0.001**
Neutral vs. Violation	0.77 *	0.05,1.48	0.37	2.10	**0.036**
Semantics vc. Moral	1.06 **	0.31,1.81	0.38	2.78	**0.005**
**Random Effects**					
σ^2	48.33				
τ_{00} Item_number	1.22				
τ_{00} Subject	2.57				
τ_{11} Item_number.ConditionLabelViol	0.94				
τ_{11} Item_number.ConditionLabelMorSem	2.64				
τ_{11} Subject.ConditionLabelViol	1.28				
τ_{11} Subject.ConditionLabelMorSem	0.16				
ρ_{01}	-0.18				
	-0.31				
	0.79				
	0.00				
N Subject	32				
N Item_number	90				
Observations	2632				
Marginal R^2 / Conditional R^2	0.007 / NA				

* $p<0.05$ ** $p<0.01$ *** $p<0.001$

Model: Amplitude ~ Condition+(1+ Condition |Subject) +(1+Condition|Item_number)
